# Fast Homogeneous *En Bloc* Staining of Large Tissue Samples for Volume Electron Microscopy

**DOI:** 10.3389/fnana.2018.00076

**Published:** 2018-09-28

**Authors:** Christel Genoud, Benjamin Titze, Alexandra Graff-Meyer, Rainer W. Friedrich

**Affiliations:** ^1^Friedrich Miescher Institute for Biomedical Research, Basel, Switzerland; ^2^Faculty of Natural Sciences, University of Basel, Basel, Switzerland

**Keywords:** EM, protocol, BROPA, connectomics, SBEM, block-face, zebrafish, sample preparation

## Abstract

Fixation and staining of large tissue samples are critical for the acquisition of volumetric electron microscopic image datasets and the subsequent reconstruction of neuronal circuits. Efficient protocols exist for the staining of small samples, but uniform contrast is often difficult to achieve when the sample diameter exceeds a few hundred micrometers. Recently, a protocol (BROPA, brain-wide reduced-osmium staining with pyrogallol-mediated amplification) was developed that achieves homogeneous staining of the entire mouse brain but requires very long sample preparation times. By exploring modifications of this protocol we developed a substantially faster procedure, fBROPA, that allows for reliable high-quality staining of tissue blocks on the millimeter scale. Modifications of the original BROPA protocol include drastically reduced incubation times and a lead aspartate incubation to increase sample conductivity. Using this procedure, whole brains from adult zebrafish were stained within 4 days. Homogenous high-contrast staining was achieved throughout the brain. High-quality image stacks with voxel sizes of 10 × 10 × 25 nm^3^ were obtained by serial block-face imaging using an electron dose of ~15 e^−^/nm^2^. No obvious reduction in staining quality was observed in comparison to smaller samples stained by other state-of-the-art procedures. Furthermore, high-quality images with minimal charging artifacts were obtained from non-neural tissues with low membrane density. fBROPA is therefore likely to be a versatile and efficient sample preparation protocol for a wide range of applications in volume electron microscopy.

## Introduction

Volume electron microscopy (volume EM) is currently the only imaging approach that enables dense reconstructions of neuronal circuits. A current goal for large-scale projects is the acquisition of high-resolution image data from volumes up to 1 mm^3^, which may be achieved by different approaches that rely on automated sectioning and imaging (Briggman and Bock, 2012; Denk et al., 2012; Titze and Genoud, 2016). One strategy is to collect sections on a support for subsequent imaging in a scanning EM (SEM) or in a transmission EM (TEM). Alternatively, stacks of images may be acquired by serial block-face scanning electron microscopy (SBEM), a technique that repeatedly cuts and images the sample block-face in an SEM (Denk and Horstmann, 2004). For both approaches, unsliced tissue blocks must be fixed and impregnated with heavy metals. Efficient methods for *en bloc* staining of EM samples on this size scale are thus of key importance for connectomics.

*En bloc* staining methods for connectomics should ideally achieve uniform and strong impregnation of membranes with heavy metals throughout large sample blocks. This has been achieved by the rOTO (reduced osmium—thiocarbohydrazide—osmium) protocol (Seligman et al., 1966; Malick and Wilson, 1975) and its recent adaptations and modifications (Deerinck et al., 2010; Tapia et al., 2012a). The rOTO protocol achieves high membrane contrast, but staining intensity decreases with depth (Hua et al., 2015), presumably because the penetration of reagents is inefficient. Acceptable staining can usually be achieved up to 200 μm below the tissue surface, but staining of thicker samples remains challenging. Hence, new approaches are required for high-contrast staining of samples containing large neuronal circuits.

Recently, two protocols for efficient staining of larger samples have been introduced. The first protocol is based on modifications of the osmium steps and produced homogeneous and strong staining in 1 × 1 × 1 mm^3^ blocks of mouse brain tissue (Hua et al., 2015). However, we obtained variable results when applying this protocol to the brain of adult zebrafish, which is difficult to impregnate because densely packed somata and meninges form strong diffusion barriers. The second protocol, referred to as BROPA, was developed to stain entire mouse brains and uses different reagents such as formamide and pyrogallol (Mikula and Denk, 2015). Uniform staining of an entire mouse brain requires very long incubation times that result in a total protocol duration of 2–3 months. We therefore explored the possibility to modify this protocol to achieve faster staining of smaller samples.

We developed a modified BROPA protocol, referred to as “fast BROPA” (fBROPA), that achieves strong and uniform staining of samples on a millimeter scale. The procedure takes advantage of the reagents used in the BROPA protocol but uses drastically shorter incubation times and contains additional modifications. A lead aspartate (Walton, 1979) incubation step substantially increased the conductivity of the sample, which greatly facilitated SBEM imaging of sample blocks. The protocol does not include uranyl acetate, thus resolving concerns about radiation safety (Odriozola et al., 2017). The time required for the complete fBROPA staining procedure (four days) is similar to the duration of other protocols such as rOTO. We tested fBROPA on different samples including tissue from the adult zebrafish brain and mammalian intestinal organoids using SBEM. In all samples, fBROPA produced uniform staining with high contrast and conductivity. We therefore conclude that fBROPA is a promising staining method for volumetric EM applications in connectomics and other fields.

## Results

The goal of this study was to develop an *en bloc* staining protocol for reliable staining of samples on the millimeter scale. Our starting point was the BROPA protocol (Mikula and Denk, 2015), which has been developed for larger samples. In order to adapt it to smaller samples we first used the same reagents and procedures but reduced incubation times by a constant factor. Protocols were then used to stain entire brains of adult zebrafish, which have a maximal diameter of >1 mm. Brains were imaged in an SEM (Zeiss Merlin or FEI Quanta 200 FEG) in low or high vacuum. Under these conditions, intense staining with heavy metals is required to obtain high-contrast images. Moreover, highly conductive samples are required to prevent charging in high vacuum.

**Figure 1:**
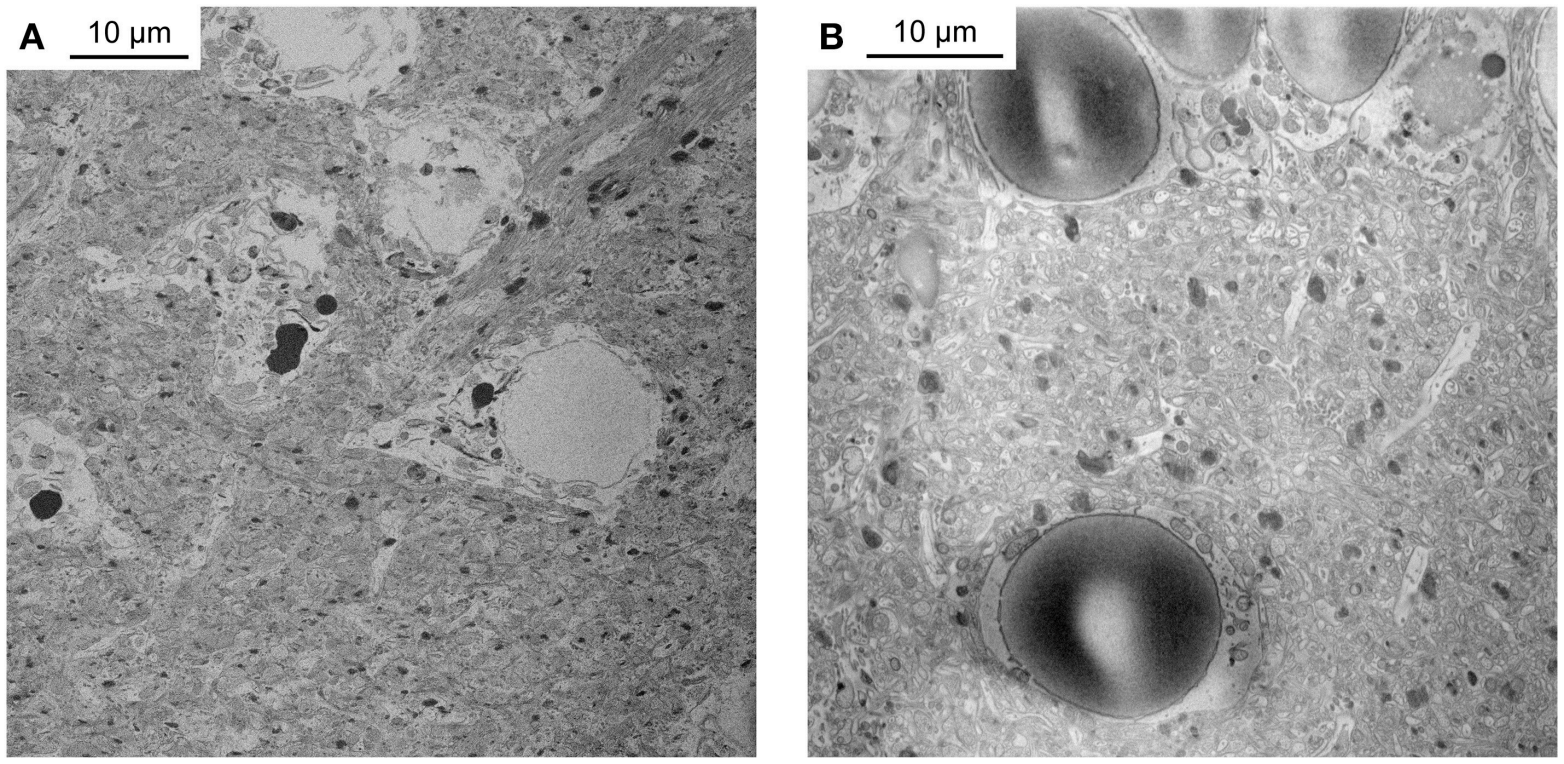
Unsuccessful staining attempts. **(A)** Telencephalon of adult zebrafish stained with a protocol that reduced incubation times of the original BROPA protocol to a total duration of 2 weeks. Note severe tissue damage. **(B)** Telencephalon of adult zebrafish stained with a protocol that reduced incubation times of the original BROPA protocol to a total duration of 5 days without lead aspartate. Note charging artifacts in nuclei and neuropil.

We first reduced all incubation times of the original BROPA protocol by a scaling factor given by the approximate length ratio of the zebrafish brain and the mouse brain, which resulted in a total duration of 2 weeks for the complete protocol. However, this approach was not successful. Although we used a fixation procedure that is known to preserve ultrastructure very well (Briggman and Denk, 2006; Deerinck et al., 2010; Tapia et al., 2012b; Hua et al., 2015; Mikula and Denk, 2015) brains showed obvious signs of damage. Staining was poor, membrane integrity was not preserved, and broken nuclear envelopes were observed (Figure 1A). Moreover, the tissue contained large empty spaces and was not sufficiently conductive to obtain high-quality SBEM images in high vacuum (Figure 1B).

**Figure 2:**
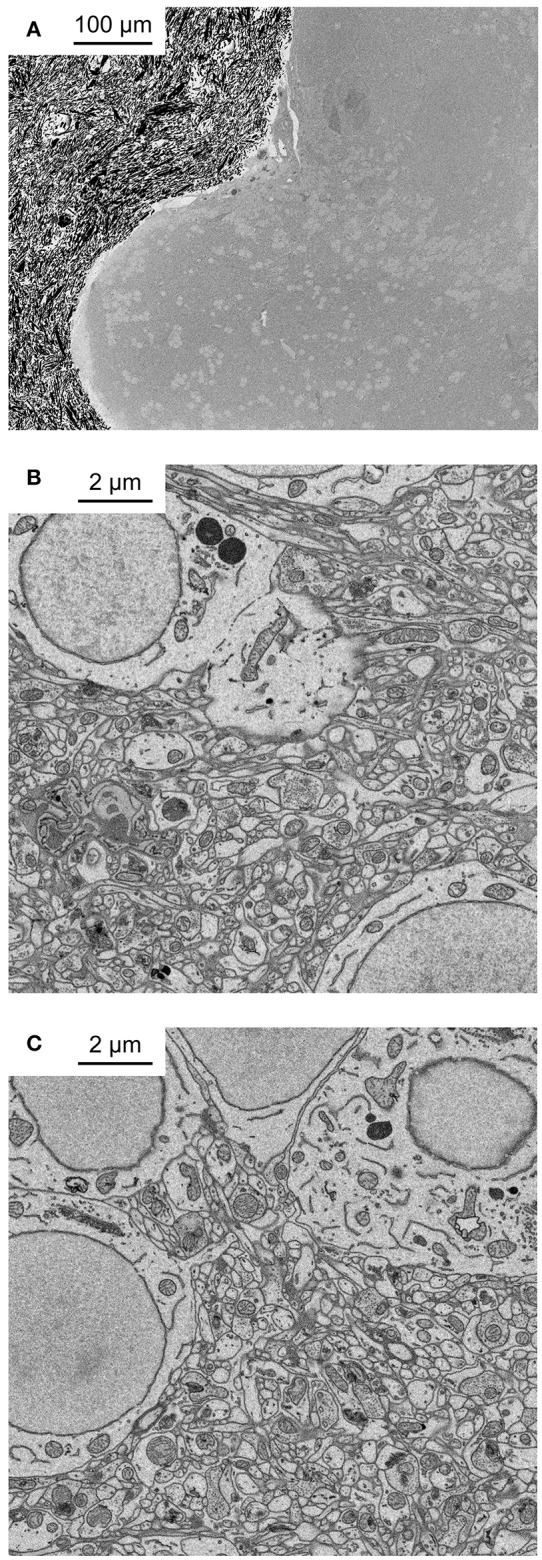
Application of fBROPA to the adult zebrafish brain. **(A)** Coronal section through the telencephalon of adult zebrafish at the level of Dp (posterior zone of the dorsal telencephalon). Note homogeneous staining. Black particles outside the tissue are silver particles in the surrounding resin to optimize conductivity. **(B)** Neuropil close to the surface. **(C)** Neuropil 300 μm below the surface.

We then varied incubation times to optimize conditions. Surprisingly, we found that shorter incubation times resulted in more intense and more homogeneous staining. Moreover, shorter incubation times eliminated obvious signs of damage and dramatically improved the preservation of tissue ultrastructure (Figure 2). To further optimize the protocol we varied the following steps and systematically analyzed staining in the telencephalon of the adult zebrafish brain:

1. Dissection and fixation. We found no obvious difference in staining between brains that were dissected in cold artificial cerebrospinal fluid (ACSF) before fixation and brains that were dissected directly in cold fixative.
2. Sucrose. We did not observe an obvious correlation between the sucrose concentration and the preservation of extracellular space (Pallotto et al., 2015). However, we observed that the proportion of extracellular space differed between brain regions. While the telencephalon contained almost no extracellular space (Figures 2B,C), substantial amounts of extracellular space were observed in the olfactory bulb.
3. Osmium incubation. Best results in the zebrafish forebrain were obtained when the durations of osmium incubations were limited to ~90 min for each incubation. This time was sufficient for reagents to diffuse throughout the forebrain and produce homogeneous staining. Longer incubation times, in contrast, may produce inhomogeneous staining. Tissue ultrastructure was well-preserved. Further observations indicated that the optimal duration of osmium incubations varies between samples. In intestinal organoids, for example, the best tissue preservation and the most homogeneous staining was obtained with an incubation time of 45 min. We therefore recommend systematic variation of this parameter when adapting the protocol to new samples.
4. Lead aspartate. In order to increase conductivity and prevent charging in the SEM (Figure 1B) we incubated samples in lead aspartate. This procedure was found to increase sample conductivity in the rOTO protocol. Consistent with this observation, we found that lead aspartate substantially increased conductivity of samples prepared by fBROPA. This step was necessary to acquire stacks of SBEM images in high vacuum.
5. Uranyl acetate. Initial attempts without lead aspartate staining produced samples that were not sufficiently conductive (Figure 1B). As a consequence, charging was severe and high-quality images in SBEM could not be obtained with beam currents >90 pA. To alleviate this problem we explored an additional incubation in uranyl acetate but did not observe an obvious increase in conductivity or image contrast. We did therefore not include a uranyl acetate incubation in the final protocol but recommend to revisit this option if problems are encountered in other samples.
6. Pyrogallol. The use of pyrogallol instead of thiocarbohydrazide (TCH) was one of the main innovations of the BROPA protocol (Mikula and Denk, 2015). We tested the option to return to TCH but abandoned this idea because pyrogallol produced substantially better results.
7. Dehydration. A time of 5–10 min was optimal to dehydrate samples without creating artifacts. It is critical that samples do not become dry at any time during dehydration. We therefore always add the next solution onto the previous one and reiterate this procedure many times to ensure that the sample is always immersed.
8. Embedding. As described previously (Wanner et al., 2016), we first embedded the sample in epoxy resin (Denk and Horstmann, 2004) and subsequently transferred it into a different resin containing silver particles. This procedure renders the sample volume around the tissue conductive and suppresses charging in the SBEM. We found that the epoxy resin for the initial embedding should be kept liquid for a longer time when samples are larger, which was achieved by variations in the formulation of the resin.

The final protocol for fBROPA is described in detail in Materials and Methods and consists of the following main steps:

Day 1: Dissection of tissue and fixation overnight.
Day 2: Incubation in reduced osmium, osmium, pyrogallol, osmium.
Day 3: Incubation in lead aspartate, dehydration, incubation in resin.
Day 4: Embedding of sample.

We used the zebrafish telencephalon to optimize the protocol because pilot experiments had shown that other sample preparation procedures often failed to produce strong and uniform staining in this brain area. One possible explanation for this observation is that the ventricle stretches as a thin sheet over the dorsal telencephalon and hinders diffusion of reagents into the tissue. Nevertheless, fBROPA resulted in uniform high-contrast staining throughout the zebrafish forebrain (Figure 2). Contrast and signal-to-noise ratio of images taken deep below the surface appeared indistinguishable from superficial images (Figures 2B,C). In some cases, contrast was even higher in deep regions as compared to superficial regions. Hence, fBROPA allows for efficient staining of large samples.

**Figure 3:**
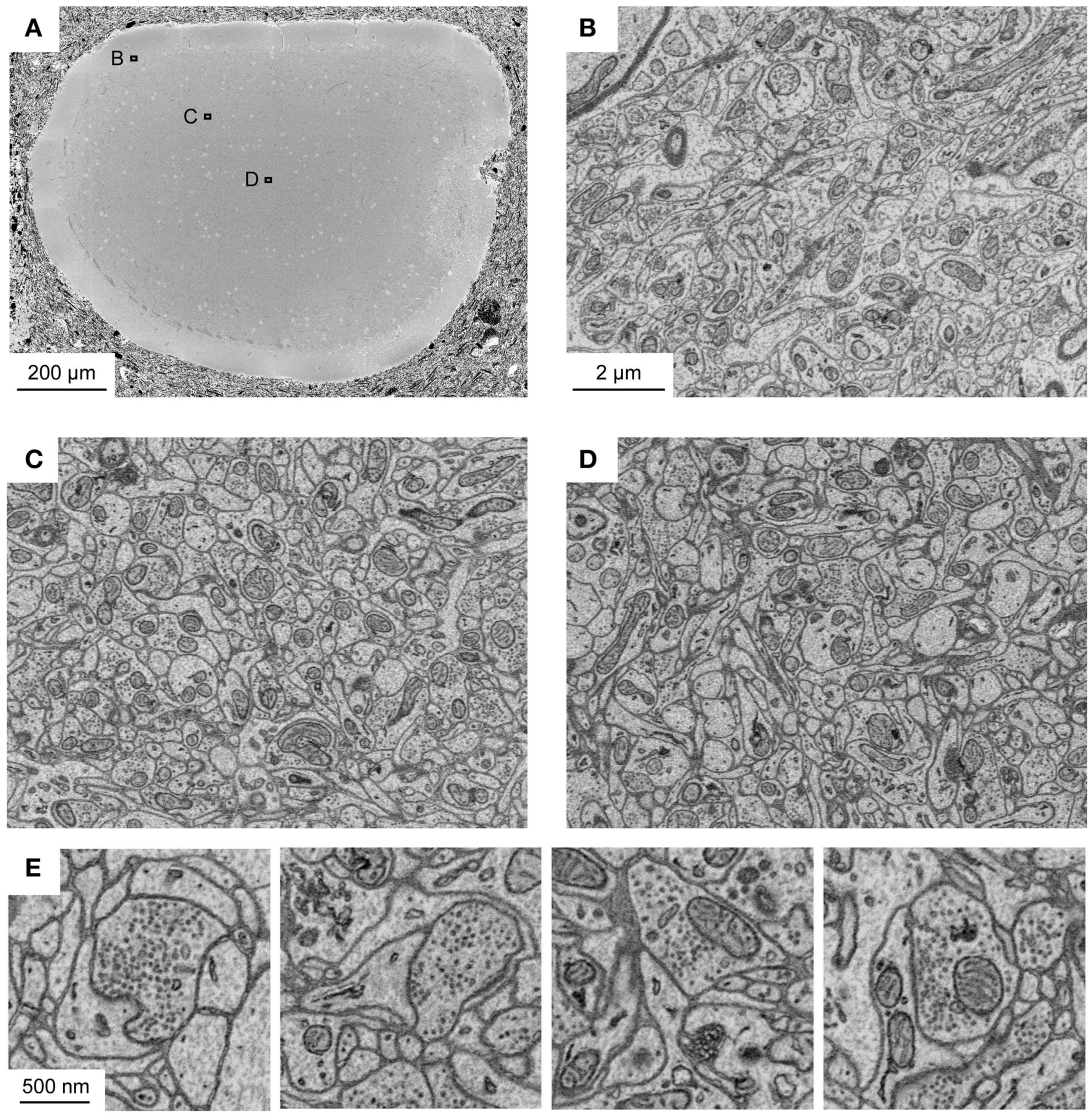
Application of fBROPA to the adult zebrafish brain. **(A)** Section through the tectum near the location where the diameter is maximal (1.1 mm). Image is a mosaic of 6 × 6 tiles. **(B–D)** Three images acquired at different depths. Approximate locations of images are indicated by outlines in **(A)**. **(E)** Examples of images showing synapses (5 nm pixel size). Vesicle pools close to the presynaptic membrane and a thickening of both membranes are visible. Synapse detection can be performed in 3D as shown in Supplementary Data S1 (movie). Note uniformly high contrast. The partial damage on the right side of the tectum occurred during dissection and is unrelated to fixation or staining.

To corroborate this conclusion we analyzed sample blocks that were cross-sectioned through the optic tectum of adult zebrafish where the diameter of the brain is maximal. The diameters of these cross-sections were ~1.1 and 0.8 mm along the long and short axes, respectively. Homogeneous staining was observed throughout (Figure 3A). High-resolution images of sub-regions in different locations demonstrated that contrast was uniformly high (Figures 3B–D). As observed with related protocols for *en bloc* staining of large volumes, synaptic vesicles could be clearly resolved while staining of postsynaptic densities was not prominent (Figure 3E). Images with high signal-to-noise ratio could be obtained at all locations using image acquisition parameters that are typically used in high-throughput SBEM (1.5 keV landing energy, 15 e^−^/nm^2^, high vacuum).

**Figure 4:**
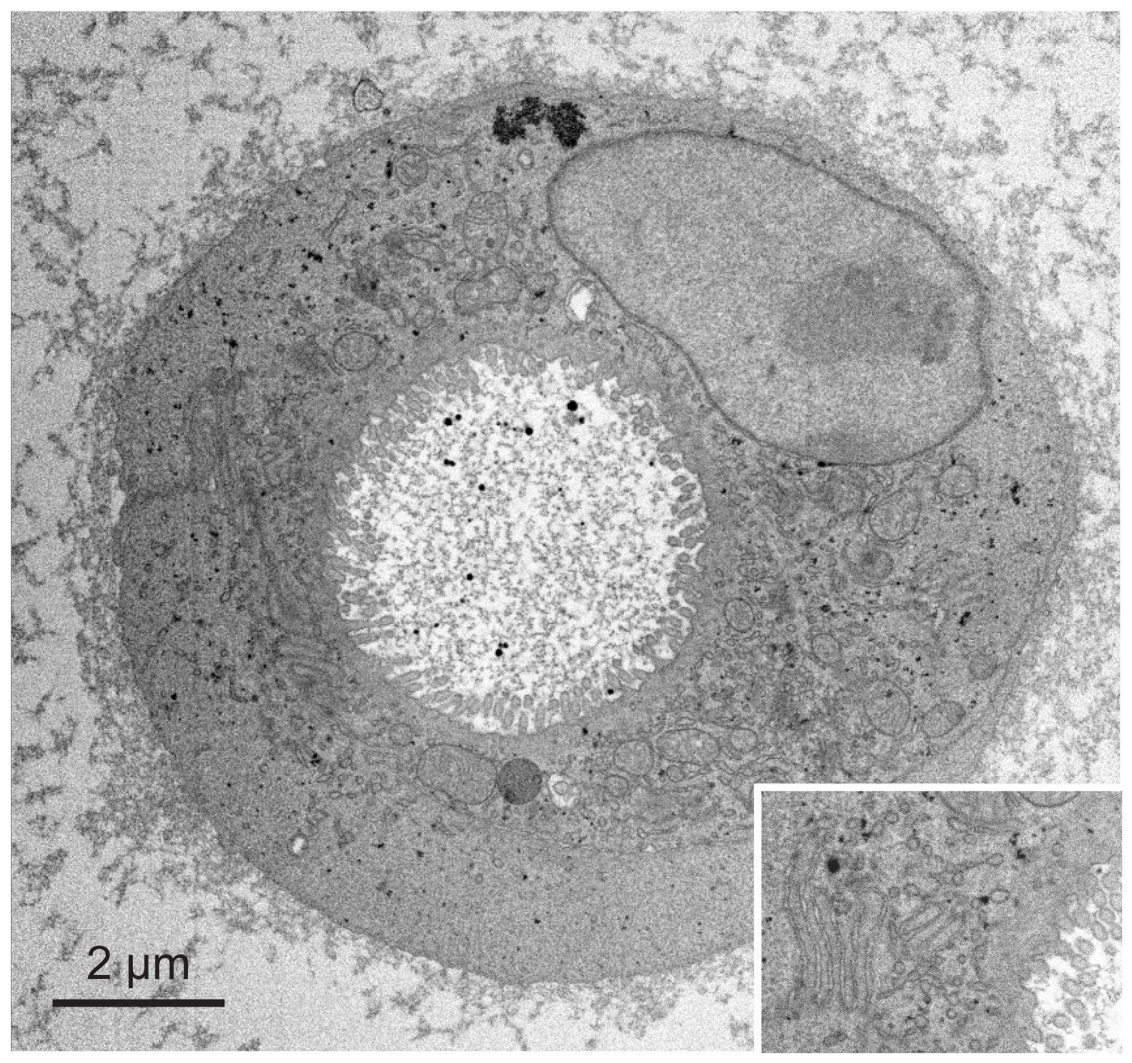
Application of fBROPA to early differentiated organoids (two cells). Insert shows details of the membrane ultrastructure between the two cells.

We next applied fBROPA to intestinal organoids that are small in volume compared to the zebrafish brain. However, these samples have a lower membrane density than brain tissue and contain relatively large sub-volumes devoid of cells. Organoid samples therefore accumulate less osmium and tend to be less conductive than brain samples. As a consequence, organoid samples are prone to charging effects and present a challenge for high-quality imaging using SBEM. We found that organoid samples prepared using fBROPA allowed for acquisition of high-resolution image stacks (3 keV landing energy, 3 nm pixel size, 50 nm section thickness; Figure 4). Charging artifacts were minimal and image quality was similar to that obtained in other samples. The acquisition of high-quality stacks at low section thickness (≤30 nm) was not possible when the incubation in lead aspartate was omitted because charging effects became too strong. These observations show that fBROPA allows for the preparation of volumetric EM samples with high contrast and conductivity from different biological sources.

## Discussion

We developed fBROPA, an *en bloc* staining method for volume EM. fBROPA is based on similar reagents and procedures as BROPA but uses substantially shorter incubation times, resulting in a total duration of four days that is similar to that of other *en bloc* staining protocols. Moreover, fBROPA includes an additional lead aspartate incubation step that substantially increases sample conductivity.

Compared to the well-established rOTO protocol, fBROPA substantially extends the volume of homogeneous staining to the millimeter range without an obvious loss in staining intensity, contrast or conductivity. The appearance of synapses is similar as in rOTO-stained tissue, with distinct synaptic vesicles and lightly stained postsynaptic densities. Homogeneous high-contrast staining of cubic millimeter samples has also been achieved by a modification of the original rOTO protocol (Hua et al., 2015). This protocol has been applied successfully to the rodent neocortex. fBROPA now provides a complementary option for *en bloc* staining of large samples that has been applied successfully to different parts of the zebrafish brain and to organoid samples. These samples present different challenges for staining procedures including diffusion barriers and low membrane density. fBROPA resulted in high contrast and conductivity in all of these samples, indicating that it may be applicable to a wide range of biological specimens. Hence, fBROPA extends the range of available options for *en bloc* staining of large EM samples. Future studies may thus further explore applications of fBROPA and compare it to alternative protocols.

Recent developments in 3D EM technology substantially increased the speed of data acquisition without loss in image quality. As a consequence, the acquisition of high-resolution image stacks covering volumes as large as a cubic millimeter appears realistic in the near future. Ultrastructural imaging of such volumes can enable the reconstruction of important neuronal circuits such as entire neocortical columns. We therefore assume that fBROPA will become a valuable method for large-scale connectomics and neuronal circuit reconstruction. Moreover, fBROPA has the potential to facilitate various applications of volumetric EM in other scientific domains.

## Materials and methods

### Reagents

Fixative: 2.5% wt/vol glutaraldehyde (16400, Electron Microscopy Sciences [EMS]) in 0.1 M cacodylate buffer (Sigma CO250) with 4% sucrose (Sigma S9378), pH 7.4. Use fresh fixative (within less than 4 hours after preparation). Add 3 ml (2.5% wt/vol) of freshly opened glutaraldehyde (25% vol/vol aqueous solution) to 15 ml 0.2 M cacodylate buffer with 1.2 g sucrose. Correct pH to 7.4, and then fill up to 30 ml with double-distilled water (ddH_2_O).

Cacodylate buffer (0.2 M stock solution): Prepare 100 ml of 0.4 M cacodylate buffer (8.56 g to 100 ml of ddH_2_O) and add ~10.8 ml of 0.2 M HCl. Adjust the pH to 7.4 and fill up to 200 ml with ddH_2_O. Can be stored at room temperature.

OsO_4_/K_4_Fe(CN)_6_ solution: 40 mM OsO_4_ (EMS 19110) with 35 mM K_4_Fe(CN)_6_ (Sigma Aldrich 60280) in 0.1 M cacodylate buffer and 2.5 M formamide (Sigma 47670). Add 2.5 ml of 4% aqueous osmium tetroxide (stock solution prepared at least 24 h in advance by dissolving 2 g of osmium tetroxide crystals in 50 ml of ddH_2_O) to 5 ml of 0.2 M cacodylate buffer. Add 0.15 g of K_4_Fe(CN)_6_ (0.147812 g for 35 mM) and 1.125 ml of formamide. Fill up to 10 ml with ddH_2_O.

OsO_4_ solution A: 40 mM OsO_4_ (EMS) in 0.1 M cacodylate buffer. Add 2.5 ml of 4% aqueous osmium tetroxide (stock solution prepared at least 24 h in advance by dissolving 2 g of osmium tetroxide crystals in 50 ml of ddH_2_O) to 5 ml of 0.2 M cacodylate buffer. Fill up to final volume of 10 ml with ddH_2_O.

OsO_4_ solution B: 40 mM OsO_4_ (EMS) in ddH_2_O. Prepare 2.5 ml of 4% aqueous osmium tetroxide (stock solution prepared at least 24 h in advance by dissolving 2 g of osmium tetroxide crystals in 50 ml of ddH_2_O) and fill up to final volume of 10 ml with ddH_2_O.

Pyrogallol (Sigma 16040), 320 mM, pH 4.1, unbuffered: Solution can be used for up to 6 weeks after preparation. Add 2.5 ml of 1.28 M stock pyrogallol solution (stock solution is obtained by adding 40.35 g in 250 ml ddH_2_O and stored in the dark at 20°C) in 7.5 ml ddH_2_O.

Walton's lead aspartate: Ensure that all solutions are kept at 60°C in a water bath and that pH is measured at this temperature. Mix 0.040 g aspartic acid (Sigma A9256) in 10 ml ddH_2_O and bring the solution to 60°C. Then add 0.066 g lead nitrate (EMS 17900) and let the solution stabilize at 60°C. Adjust pH to 5.5 (at 60°C) with 1M NaOH (~350 μl). Keep solution at 60°C throughout.

Uranyl acetate solution (1% wt/vol in ddH_2_O): Add 0.2 g of uranyl acetate powder to 20 ml of ddH_2_O, and agitate gently until solution is transparent.

Durcupan resin (Knott et al., 2009): Mix 33.3 g of resin A/M (Sigma 44611), 33.3 g of hardener B (Sigma 44612) and 1 g of hardener D (Sigma 44614) into a plastic pouring flask. Stir continuously with magnetic stirrer for at least 30 min. Add 16 drops of DMP-30 (tris-(dimethylaminomethyl)phenol, EMS 13600), and stir for a further 10 min.

Durcupan resin (Deerinck et al., 2017): Mix 11.4 g part A/M (Sigma 44611), 10 g part B (Sigma 44612) and 0.3 g part C (Sigma 44613) into a plastic pouring flask. Stir continuously with magnetic stirrer for at least 30 min. Add 0.05-0.1 g part D (Sigma 44614). For both recipes of Durcupan, the EMS products can also be used.

Epoxy “SERVA” resin (Denk and Horstmann, 2004): Mix 11.1 g of glycid ether (SERVA Electrophoresis GmbH 21045), 6.19 g of dodecenylsuccinic anhydride (DDSA, SERVA Electrophoresis GmbH 20755), and 6.25 g of methyl nadic anhydride (MNA, SERVA Electrophoresis GmbH 29451) into a plastic pouring flask. Stir continuously with magnetic stirrer for at least 30 min. Add 0.325 ml of benzyldimethylamine (BDMA, SERVA Electrophoresis GmbH 14835), and stir for a further 10 min.

Epoxy “EMbed812” resin: Mix 20 ml of “EMbed812” (EMS kit 14120), 16 ml of DDSA (EMS kit 14120), and 8 ml of MNA (EMS kit 14120) into a plastic pouring flask. Stir continuously with magnetic stirrer for at least 30 min. Add 0.75 ml of DMP-30 (tris-(dimethylaminomethyl)phenol, EMS 13600), and stir for a further 10 min.

### fBROPA protocol for adult zebrafish brain

1. Dissect brain in ice-cold, precarbogenated ACSF as described (Zhu et al., 2012).
2. Immerse in fixative for 1 hour at room temperature and then overnight at 4°C.
3. The next morning, replace the fixative by 0.1 M cacodylate buffer with 4% sucrose, pH 7.4. Samples can be stored in this buffer for at least seven days when the medium is changed every other day.
4. Stain in freshly prepared OsO_4_/K_4_Fe(CN)_6_ solution (40 mM OsO_4_ with 35 mM K_4_Fe(CN)_6_ in 0.1 M cacodylate buffer and 2.5 M formamide) at room temperature **for 90 min**.
5. Stain in OsO_4_ solution A (40 mM OsO_4_ in 0.1 M cacodylate buffer), pH 7.4, at room temperature for **90 min**. Do not rinse between steps 4 and 5.
6. Wash at least 3 × 5 min with 0.1 M cacodylate buffer, pH 7.4. Repeat rinsing until solution remains clear for 5 min.
7. Place sample in 320 mM pyrogallol, pH 4.1, unbuffered, in ddH_2_O, **for 30 min**.
8. Wash at least 3 × 5 min with 0.1 M cacodylate buffer, pH 7.4. Repeat rinsing until solution remains clear for 5 min.
9. Stain in in OsO_4_ solution B (40 mM OsO_4_ in ddH_2_O) at room temp for **90 min**.
10. Store in ddH_2_O at 4°C overnight.
11. Wash 3 × 5 min in ddH_2_O at room temperature.
12. Immerse in Walton's lead aspartate solution at 60°C for **60 min**.
13. Wash 3 × 5 min in ddH_2_O at room temperature.
14. Dehydrate in graded EtOH balanced with water (10%, 25%, 50%, 75%, 2 × 100%) at room temperature or on ice.
15. Depending on the resin used, the sample may be incubated in 100% propylene oxide twice for 10 min. This step is optional and may be used if the resin does not penetrate throughout the tissue. Otherwise, we recommend avoiding this step because it can wash out reagents.
16. Immerse the sample in 50% resin−50% propylene oxide, or in 50% resin−50% ethanol, for at least 120 min. This step can be extended overnight. We successfully used four recipes of resin (see Reagents for details). Ensure that the resin penetrates evenly through the sample.
17. Immerse the sample in 100% resin overnight before embedding.
18. If samples need to be oriented on the stub used for SBEM imaging, transfer samples to the stub in liquid resin. Superglue can be used to glue the sample in liquid resin onto the stub. If the sample is to be embedded in silver-containing resin for high conductivity, proceed as described in (Wanner et al., 2016).
19. Cure the resin in the oven at 60°C for at least 48 h.
20. Trim sample in an ultramicrotome to prepare the block surface for the SBEM (Wanner et al., 2016).

As recommended in Mikula and Denk (2015), tubes should be changed for each staining step.

All experiments were approved by the Veterinary Department of the Canton Basel-Stadt (Switzerland).

### Imaging

Images were acquired on a Zeiss Merlin SEM (Zeiss, Oberkochen, Germany) and on a Quanta 200 VP-FEG (FEI, Eindhoven, Netherlands; now ThermoFisher Scientific). Both microscopes were equipped with an automated ultramicrotome inside the vacuum chamber for SBEM (3View; Gatan, Pleasanton, CA, USA; now ThermoFisher Scientific). On the Zeiss Merlin, image acquisition was controlled by *SBEMimage*, an open-source software for image acquisition in SBEM (Titze et al., 2018). Images were acquired with a landing energy of 1.5 keV in analytical mode. Other imaging parameters were chosen to maintain an electron dose of 15 e^−^/nm^2^ (beam current: 300 pA, pixel dwell time: 0.8 μs, pixel size: 10 × 10 nm^2^). These conditions were used to acquire all high-resolution images from the zebrafish brain. Images from intestinal organoids were acquired on the Quanta 200 with a landing energy of 3 keV, a pixel size of 3 × 3 nm^2^, and a pixel dwell time of 2 μs.

## Author contributions

All authors contributed to conception and design of the method. AG-M, BT, and CG performed the experiments and imaging. CG wrote the first draft of the manuscript. RF wrote the final manuscript. All authors contributed to manuscript revision, read, and approved the submitted version.

### Conflict of interest statement

The authors declare that the research was conducted in the absence of any commercial or financial relationships that could be construed as a potential conflict of interest.

## Supplementary material

Supplementary Data S1 Movie of an image stack from the adult zebrafish brain prepared using the fBROPA protocol. Field of view is 8 × 8 μm^2^. Original voxel size is 10 × 10 × 30 nm^3^.
